# Supplementary material for: Determinants of WHO recommended COVID-19 prevention measures among pregnant women attending antenatal care during the third wave of COVID-19 in eastern Ethiopia, 2021
Source: PLoS One. 2023 May 25;18(5):e0284643. doi: 10.1371/journal.pone.0284643 (PMC10212107; doi:10.1371/journal.pone.0284643)
Supplement: S1 File — (DOCX) [file pone.0284643.s002.docx]

**Annex: Questionnaire for Assessing Prevention Practice and Determinants of WHO Recommended Covid-19 Prevention Measures Among Pregnant Women Attending Antenatal Care During the Third Wave of COVID-19 in Eastern Ethiopia, 2021**

**Part I: Sociodemographic characteristics**

| DATE____/_____/. | | |
| --- | --- | --- |
| **Part one: Sociodemographic Characteristics** | | |
| S/N | QUESTIONS | RESPONSES |
| 001 | Age in years | _________ in years |
| 002 | Residence | 1. Urban 2. Rural |
| 003 | Average monthly income | _________ ETB |
| 004 | Educational status of women | 1. Unable to read and write 2. Primary education 3. Secondary education 4. Above secondary school |
| 005 | Educational status of Husband | 1. Unable to read and write 2. Primary education 3. Secondary education 4. Above secondary school |
| 006 | Family size | _________ |
| 007 | Occupation | 1. Student 2. House wife 3. Merchant 4. Civil servant |
| 008 | Age at first marriage | _________ in years |
| 009 | Number of Children | _________ |
| 010 | Number of pregnancies | _________ |

**Part II: WHO Recommended COVID-19 preventive measure related variables (knowledge, attitude, and practice)**

| **Section A: Knowledge towards WHO Recommended COVID-19 preventive measures** | | |
| --- | --- | --- |
| S/N | QUESTIONS | RESPONSES |
| 001 | Do you know washing hands for 20 seconds can prevent the virus? | 1. Yes  2. No |
| 002 | Do you know that sneezing or coughing into arm/elbow can prevent spread of  virus? | 1. Yes  2. No |
| 003 | Do you know COVID-19 virus can be transmitted by shaking hands? | 1. Yes  2. No |
| 004 | Do you know maintaining safe distance at least one meter can protect from  the virus? | 1. Yes  2. No |
| 005 | Do you know touching face can transfer the virus? | 1. Yes  2. No |
| 006 | Do you know staying at home can decrease the chance of getting infected? | 1. Yes  2. No |
| 007 | Do you know wearing the mask can prevent the virus? | 1. Yes  2. No |
| **Section B: Practice towards WHO Recommended COVID-19 preventive measures** | | |
| 001 | Do you wash hands for 20 seconds? | 1. Yes  2. No |
| 002 | Do you sneeze/cough into arm/elbow? | 1. Yes  2. No |
| 003 | Do you avoid shaking hands? | 1. Yes  2. No |
| 004 | Do you maintain a social distance at least one meter? | 1. Yes  2. No |
| 005 | Do you avoid touching your face? | 1. Yes  2. No |
| 006 | Do you stay at home quite often? | 1. Yes  2. No |
| 007 | Do you use face mask? | 1. Yes  2. No |
