# Supplementary material for: Determinants of WHO recommended COVID-19 prevention measures among pregnant women attending antenatal care during the third wave of COVID-19 in eastern Ethiopia, 2021
Source: PLoS One. 2023 May 25;18(5):e0284643. doi: 10.1371/journal.pone.0284643 (PMC10212107; doi:10.1371/journal.pone.0284643)
Supplement: S2 File — (DOCX) [file pone.0284643.s003.docx]

[DataSet1] C:\Users\As\Desktop\COVID PAPER\prevention practice toward COVID-19 ANC\Dove_ANC_covid19\Prevention Practice covid ANC.sav222.sav

**Frequency Table**

| **do you know that washing your handsfor 20 sec prevent the virus?** | | | | | |
| --- | --- | --- | --- | --- | --- |
|  | | Frequency | Percent | Valid Percent | Cumulative Percent |
| Valid | Yes | 379 | 89.8 | 89.8 | 89.8 |
|  | No | 43 | 10.2 | 10.2 | 100.0 |
|  | Total | 422 | 100.0 | 100.0 |  |

| **do you wash your hands for 20 seconds?** | | | | | |
| --- | --- | --- | --- | --- | --- |
|  | | Frequency | Percent | Valid Percent | Cumulative Percent |
| Valid | Yes | 307 | 72.7 | 72.7 | 72.7 |
|  | No | 115 | 27.3 | 27.3 | 100.0 |
|  | Total | 422 | 100.0 | 100.0 |  |

| **do you know that sneezing or coughing into your arm /elbow can prevent the spread of the virs** | | | | | |
| --- | --- | --- | --- | --- | --- |
|  | | Frequency | Percent | Valid Percent | Cumulative Percent |
| Valid | Yes | 371 | 87.9 | 87.9 | 87.9 |
|  | No | 51 | 12.1 | 12.1 | 100.0 |
|  | Total | 422 | 100.0 | 100.0 |  |

| **do you sneeze into your arm/elbow?** | | | | | |
| --- | --- | --- | --- | --- | --- |
|  | | Frequency | Percent | Valid Percent | Cumulative Percent |
| Valid | Yes | 311 | 73.7 | 73.7 | 73.7 |
|  | No | 111 | 26.3 | 26.3 | 100.0 |
|  | Total | 422 | 100.0 | 100.0 |  |

| **do you know that the virus can be transmitted by shaking hands?** | | | | | |
| --- | --- | --- | --- | --- | --- |
|  | | Frequency | Percent | Valid Percent | Cumulative Percent |
| Valid | Yes | 370 | 87.7 | 87.7 | 87.7 |
|  | No | 52 | 12.3 | 12.3 | 100.0 |
|  | Total | 422 | 100.0 | 100.0 |  |

| **do you avoid shaking hands whilst greeting** | | | | | |
| --- | --- | --- | --- | --- | --- |
|  | | Frequency | Percent | Valid Percent | Cumulative Percent |
| Valid | Yes | 240 | 56.9 | 56.9 | 56.9 |
|  | No | 182 | 43.1 | 43.1 | 100.0 |
|  | Total | 422 | 100.0 | 100.0 |  |

| **do you know that you should maintain a safe distance of at least one meter while sitting withother people** | | | | | |
| --- | --- | --- | --- | --- | --- |
|  | | Frequency | Percent | Valid Percent | Cumulative Percent |
| Valid | Yes | 358 | 84.8 | 84.8 | 84.8 |
|  | No | 64 | 15.2 | 15.2 | 100.0 |
|  | Total | 422 | 100.0 | 100.0 |  |

| **do you maintain -a distance of at least one meter?** | | | | | |
| --- | --- | --- | --- | --- | --- |
|  | | Frequency | Percent | Valid Percent | Cumulative Percent |
| Valid | Yes | 251 | 59.5 | 59.5 | 59.5 |
|  | No | 171 | 40.5 | 40.5 | 100.0 |
|  | Total | 422 | 100.0 | 100.0 |  |

| **do you know that touching your face can transfer virus?** | | | | | |
| --- | --- | --- | --- | --- | --- |
|  | | Frequency | Percent | Valid Percent | Cumulative Percent |
| Valid | Yes | 358 | 84.8 | 84.8 | 84.8 |
|  | No | 64 | 15.2 | 15.2 | 100.0 |
|  | Total | 422 | 100.0 | 100.0 |  |

| **do you avoid touching your face?** | | | | | |
| --- | --- | --- | --- | --- | --- |
|  | | Frequency | Percent | Valid Percent | Cumulative Percent |
| Valid | Yes | 242 | 57.3 | 57.3 | 57.3 |
|  | No | 180 | 42.7 | 42.7 | 100.0 |
|  | Total | 422 | 100.0 | 100.0 |  |

| **do you know that staying at home can decrease the chance of getting infected?** | | | | | |
| --- | --- | --- | --- | --- | --- |
|  | | Frequency | Percent | Valid Percent | Cumulative Percent |
| Valid | Yes | 331 | 78.4 | 78.4 | 78.4 |
|  | No | 91 | 21.6 | 21.6 | 100.0 |
|  | Total | 422 | 100.0 | 100.0 |  |
| **do you stay at home quite often?** | | | | | |
|  | | Frequency | Percent | Valid Percent | Cumulative Percent |
| Valid | Yes | 187 | 44.3 | 44.3 | 44.3 |
|  | No | 235 | 55.7 | 55.7 | 100.0 |
|  | Total | 422 | 100.0 | 100.0 |  |

| **do you know that wearing a mask can prevent the virus?** | | | | | |
| --- | --- | --- | --- | --- | --- |
|  | | Frequency | Percent | Valid Percent | Cumulative Percent |
| Valid | Yes | 398 | 94.3 | 94.3 | 94.3 |
|  | No | 24 | 5.7 | 5.7 | 100.0 |
|  | Total | 422 | 100.0 | 100.0 |  |

| **do you use a mask?** | | | | | |
| --- | --- | --- | --- | --- | --- |
|  | | Frequency | Percent | Valid Percent | Cumulative Percent |
| Valid | Yes | 349 | 82.7 | 82.7 | 82.7 |
|  | No | 73 | 17.3 | 17.3 | 100.0 |
|  | Total | 422 | 100.0 | 100.0 |  |

| **did you take Covid-19 vaccine?** | | | | | |
| --- | --- | --- | --- | --- | --- |
|  | | Frequency | Percent | Valid Percent | Cumulative Percent |
| Valid | Yes | 175 | 41.5 | 41.5 | 41.5 |
|  | No | 247 | 58.5 | 58.5 | 100.0 |
|  | Total | 422 | 100.0 | 100.0 |  |

| **Level_of_knowledge** | | | | | |
| --- | --- | --- | --- | --- | --- |
|  | | Frequency | Percent | Valid Percent | Cumulative Percent |
| Valid | Good knowledge | 232 | 55.0 | 55.0 | 55.0 |
|  | Poor Knowledge | 190 | 45.0 | 45.0 | 100.0 |
|  | Total | 422 | 100.0 | 100.0 |  |

| **Level_of_Practice** | | | | | |
| --- | --- | --- | --- | --- | --- |
|  | | Frequency | Percent | Valid Percent | Cumulative Percent |
| Valid | Good Practice | 260 | 61.6 | 61.6 | 61.6 |
|  | Poor Practice | 162 | 38.4 | 38.4 | 100.0 |
|  | Total | 422 | 100.0 | 100.0 |  |
