# Supplementary material for: Determinants of WHO recommended COVID-19 prevention measures among pregnant women attending antenatal care during the third wave of COVID-19 in eastern Ethiopia, 2021
Source: PLoS One. 2023 May 25;18(5):e0284643. doi: 10.1371/journal.pone.0284643 (PMC10212107; doi:10.1371/journal.pone.0284643)
Supplement: Attachment — Submitted filename: Comments .pdf [file pone.0284643.s004.pdf]

Comments to the Authors

This manuscript described "Prevention practice and determinants of WHO recommended COVID-19 prevention measures among pregnant women attending antenatal care during the third wave of COVID-19 in eastern Ethiopia, 2021: an institutional-based cross-sectional study**"** which is an important issue to assess the practice and predictors of COVID-19 prevention measures among more risky groups pregnant women particularly in the study area. Despite the interesting scope of the research, the manuscript needs minor revision by considering the comments written below.

Title: The title is appropriate, but needs modification try to make SMART (it's 30 words)

Abstract

A. Result: Please what is your outcome of interest? Good practice Vs Poor practice or WHO recommended COVID-19 preventive measures practice? Please include all significant predictors of practice of COVID-19 preventive measures

B. Conclusion and recommendation: Make it short and correlate with the findings of the study.

- **Introduction**

1. What makes your study differ from others; because your study also assessed level of practice towards COVID-19 preventive measures?
2. Please add recent study like…. Compliance of COVID-19 preventive measures among pregnant women….in Ethiopia

- **Methods**

1. Is this study conducted at all 14 health facilities? If so, indicate the study was conducted at all 14 health facilities in Harari region ….. Under study setting section.
2. Eligibility Criteria: ……. and **<u>had the willingness to participate</u>** in the study were included. Do you think that you have conducted with correct sampling technique? …. This indicated that those pregnant women who were selected using the sampling technique and who were refused to participate were excluded in your study. That's wrong; there is selection bias.

3. Why determined your sample size with 50%? Why not used studies conducted in Southern Ethiopia, Debre Berhan ….?
4. Write your Kth interval
5. Operational definitions and measurements: put your reference for your operational definition and write how you value respondents' answer ''***for all knowledge, and practice questions participants who answered the "correct answer" or "Yes" were earned a "1 score", whereas participants who gave the "wrong answer" or "No" were earned a "0 score***" separately.

- **Result**
  - A. Please write all identified factors under section ''Factors associated with WHO recommended COVID-19 Prevention practice''
  - B. L196: The level of prevention practice among pregnant women was 61.6%… It's not clear; please indicate the level of practice as Good Vs Poor practice???
- **Discussion**
  - A. The discussion substantially needs rewriting; clear and logical justification
  - B. Please use recent studies to compare and contrast…..
- **Conclusion:**

- Needs to be more punchy and to the point/ findings.
