# Supplementary material for: Determinants of WHO recommended COVID-19 prevention measures among pregnant women attending antenatal care during the third wave of COVID-19 in eastern Ethiopia, 2021
Source: PLoS One. 2023 May 25;18(5):e0284643. doi: 10.1371/journal.pone.0284643 (PMC10212107; doi:10.1371/journal.pone.0284643)
Supplement: Attachment — Submitted filename: Response to Reviewers.docx [file pone.0284643.s005.docx]

**Point-By-Point Feedback Based on Comments and Suggestions**

**Reviewer 1**

|  | **Comments** | **Authors’ response** | **Page No.** |
| --- | --- | --- | --- |
| 1. | This manuscript described “Prevention practice and determinants of WHO recommended COVID-19 prevention measures among pregnant women attending antenatal care during the third wave of COVID-19 in eastern Ethiopia, 2021: an institutional-based cross-sectional study” which is an important issue to assess the practice and predictors of COVID-19 prevention measures among more risky groups pregnant women particularly in the study area. Despite the interesting scope of the research, the manuscript needs minor revision by considering the comments written below. | - Thank you for your response |  |
|  | Title: The title is appropriate, but needs modification try to make SMART (it’s 30 words) | - Thank you for your constructive comment, as per your suggestion we have made modification. | #1 |
|  | Abstract A. Result: Please what is your outcome of interest? Good practice Vs Poor practice or WHO recommended COVID-19 preventive measures practice?  Please include all significant predictors of practice of COVID-19 preventive measures. | - Our outcome of interest was to asses the status of WHO COVID-19 recommended prevention practice (good Vs poor) among pregnant women. - We have included all significant predictors of practice of COVID-19 preventive measures. | #2 |
|  | B. Conclusion and recommendation: Make it short and correlate with the findings of the study. | - Thank you for your constructive comments, we have addressed these issues as per your suggestion. | #2,3 |
|  | ¬ Introduction 1. What makes your study differ from others; because your study also assessed level of practice towards COVID-19 preventive measures?  2. Please add recent study like…. Compliance of COVID-19 preventive measures among pregnant women….in Ethiopia | - Thank you for your question, what makes our study different from other studies is that, there is no prior study conducted in the study area, this study was conducted during the third wave, and also there are novel findings which were not reported in other similar studies.   - As per your we have added additional study. | #5 |
|  | ¬ Methods 1. Is this study conducted at all 14 health facilities? If so, indicate the study was conducted at all 14 health facilities in Harari region ….. Under study setting section.  2. Eligibility Criteria: ……. and had the willingness to participate in the study were included.  Do you think that you have conducted with correct sampling technique? …. This indicated that those pregnant women who were selected using the sampling technique and who were refused to participate were excluded in your study. That’s wrong; there is selection bias. 3. Why determined your sample size with 50%? Why not used studies conducted in Southern Ethiopia, Debre Berhan ….? 4. Write your Kth interval  5. Operational definitions and measurements: put your reference for your operational definition and write how you value respondents’ answer ‘‘for all knowledge, and practice questions participants who answered the “correct answer” or “Yes” were earned a “1 score”, whereas participants who gave the “wrong answer” or “No” were earned a “0 score” separately. | 1- Thank you for your constructive comment, as you have suggested, we have added another additional statement “all 14 health facilities in Harari region were included in the study….. Under study setting section.”  2. Willingness to participate in the study means only for those women who were selected by random sampling method, it does not mean that selection only by their own willingness.   - When we say women “who were refused to participate were excluded in your study” means only for those women who were selected by random sample, this means they were considered as non response rate, fortunately we didn’t encountered such refusal.   3. yes indeed we have considered to use the study you have indicated but to get the maximum sample size we have used 50%. This allows as to get relatively a higher precision.  4. thank you, we have written the kth interval as you have suggested.  5. Thank you for your comment, we have cited the reference as you have suggested. |  |
|  | ¬ Result A. Please write all identified factors under section ‘‘Factors associated with WHO recommended COVID-19 Prevention practice’’ B. L196: The level of prevention practice among pregnant women was 61.6%… It’s not clear; please indicate the level of practice as Good Vs Poor practice???  ¬ Discussion A. The discussion substantially needs rewriting; clear and logical justification B. Please use recent studies to compare and contrast….. ¬ Conclusion: - Needs to be more punchy and to the point/ findings. | - A. Thank you, we have added citations, and also, we have added some descriptions as per your suggestion in lines “89-91”  - B. This is to mean the overall 61.6% of pregnant women had good level of WHO recommended COVID-19 prevention practice and we have rewritten this in the result part.    Discussion  A. Thank you very much, we have addressed according to your suggestions.  B. we have cited recent study.  Conclusion   - Thank you for your constructive comments, we have rewrite as per your suggestion. | # 11-15 |
|  | ○ The operational definitions are odd. Comparing individuals’ scores to the study group’s mean does not give you good information about having good or poor knowledge. A more useful comparison would be the group’s mean compared to a larger population’s mean. That way, you could say the population in Harar, Ethiopia had better or worse knowledge than populations in other areas of Ethiopia, or in Sub-Saharan Africa, or anywhere else. Another more useful measurement would be to set a threshold score that is good based on previous literature instead of using the group mean to set the threshold. |  |  |
|  | o Measurement used is not really sufficient to measure the intended outcome variables like knowledge, attitude and level of practice. | - Thank you for your comment, we used the common way of studying KAP studies, also there were similar studies that used this type of measurement.  - Al-Hanawi MK, Angawi K, Alshareef N, Qattan AM, Helmy HZ, Abudawood Y, Alqurashi M, Kattan WM, Kadasah NA, Chirwa GC, Alsharqi O. Knowledge, attitude and practice toward COVID-19 among the public in the Kingdom of Saudi Arabia: a cross-sectional study. Frontiers in public health. 2020 May 27;8:217.  - Siddiqui AA, Alshammary F, Amin J, Rathore HA, Hassan I, Ilyas M, Alam MK. Knowledge and practice regarding prevention of COVID-19 among the Saudi Arabian population. Work. 2020;66(4):767-775. doi: 10.3233/WOR-203223. PMID: 32925138. |  |

**Reviewer 2**

|  | **Comments** | **Authors’ response** | **Page No.** |
| --- | --- | --- | --- |
| 1. | Abstract Well written | - Thank you for your constructive comments. |  |
| `2. | Background - Lines 61-65: Could the authors kindly update the statistics!  - Lines 82-86: It would be great to compare the severity of COVID-19 statistics for pregnant women in African during the first and second waves! | - Thank you for your comment, we have used the data that was presented by WHO during our data collection period “In Africa, there have been more than 6 million confirmed COVID-19 cases, with 149,041 deaths.[6] Between 3 January 2020 and 18 October 2021, there were more than 300 thousand confirmed cases of COVID-19 in Ethiopia, with 6,197 deaths”. It is not appropriate to use the data or statistics released after our study period (October – November, 2021)   - Thank you for your comment, At the time of the present study, there has been no such study conducted in Africa to compare the severity of COVID-19 among pregnants in the first and second wave.   the common way of studying the KAP studies, also there were similar studies that used these types of measurements. |  |
| 3. | Methods - Lines 167-172: What informed the authors decision to use the mean as a cut off points for good and poor knowledge/practice? That has to been backed with scientific evidence. | - Thank you for your comment, the reason why we have decided to use the mean score as a cut off point was based on other similar published studies.  -Here are some of the relevant literature that used a similar method: **-**  - Aklil MB, Temesgan WZ. Knowledge and Attitude towards COVID-19 Vaccination and Associated Factors among College Students in Northwest Ethiopia, 2021. *Health Services Research and Managerial Epidemiology*. January 2022. doi:[10.1177/23333928221098903](https://doi.org/10.1177/23333928221098903)  - Endriyas M, Kawza A, Alano A, Hussen M, Mekonnen E, et al. (2021) Knowledge and attitude towards COVID-19 and its prevention in selected ten towns of SNNP Region, Ethiopia: Cross-sectional survey. PLOS ONE 16(8): e0255884. <https://doi.org/10.1371/journal.pone.0255884>  - Getawa S, Aynalem M, Bayleyegn B, Adane T (2022) Knowledge, attitude and practice towards COVID-19 among secondary school students in Gondar town, Northwest Ethiopia. PLOS ONE 17(5): e0268084. <https://doi.org/10.1371/journal.pone.0268084>  - Al-Hanawi MK, Angawi K, Alshareef N, Qattan AM, Helmy HZ, Abudawood Y, Alqurashi M, Kattan WM, Kadasah NA, Chirwa GC, Alsharqi O. Knowledge, attitude and practice toward COVID-19 among the public in the Kingdom of Saudi Arabia: a cross-sectional study. Frontiers in public health. 2020 May 27;8:217.  - Siddiqui AA, Alshammary F, Amin J, Rathore HA, Hassan I, Ilyas M, Alam MK. Knowledge and practice regarding prevention of COVID-19 among the Saudi Arabian population. Work. 2020;66(4):767-775. doi: 10.3233/WOR-203223. PMID: 32925138. |  |
| 4. | Results - Lines 208-217: Kindly report all odds ratios with their decimal points as in table 4. - Lines 216-217: The interpretation is not accurate. It is 76% lower odds BUT NOT 76% times………. | - Thank you very much for your suggestion, we have made changes accordingly. | #12 |
| 5. | - Authors should be consistent with the use of decimal points on the table and throughout the manuscript. They should either stick to one or two decimal points but not use both. | - Thank you, we have made consistent the percentage decimals as per your suggestion. | #9-13 |
| 6. | Discussion - Lines 250-252: Provide a citation to back the speculation. - Lines 258-259: Provide a citation to back the speculation. - Lines 262-263: The interpretation is incorrect. Kindly refer to table 4 to write the right interpretation. - Lines 263-265: Provide a citation to back the speculation. - Lines 267-271: Provide citations to back the speculations. | - Thank you and we highly appreciate your point of view. however, all the lines you have mentioned from the discussion part are our novel findings, which were not reported in other similar studies. That is why we kept as it is with out citation. |  |
| 7. | Since this was an institutional-based cross-sectional study, it can only be generalized as such. This should be stated as one of the weaknesses of the study. | - Thank you for your constructive comment, we have included this idea under the limitation part of the study. | #16 |
