# Supplementary material for: Determinants of WHO recommended COVID-19 prevention measures among pregnant women attending antenatal care during the third wave of COVID-19 in eastern Ethiopia, 2021
Source: PLoS One. 2023 May 25;18(5):e0284643. doi: 10.1371/journal.pone.0284643 (PMC10212107; doi:10.1371/journal.pone.0284643)
Supplement: Attachment — Submitted filename: Response to Reviewers.docx [file pone.0284643.s006.docx]

**Point-By-Point Feedback Based on Comments and Suggestions**

**Editor**

|  | **Comments** | **Authors’ response** | **Page No.** |
| --- | --- | --- | --- |
| 1. | I agree with the reviewers that the manuscript needs further revision  Overall, the manuscript needs language editing. Please obtain language editing assistance before you submit the revised manuscript. | - Thank you very much for your constructive feedback, we have checked again the language and grammatical flow of the manuscript throughout the paper, additionally, we have used software and English language professionals. | #1 |
| 2 | Your research paper’s title is 28 words long. If possible, shorten it. | - Thank you for your constructive feedback, we have shortened the topic from 28 words to 23 words, as per your suggestion. | #9 |
| 3. | In the financial disclosure, the authors state that Harar Health Sciences College played a role in the study design, data collection and analysis, decision to publish, or manuscript preparation.   a. What were the interests and positions of the funder in the study? b. How did this impact the data collection, analysis, and interpretation?      c. Whose views are reflected in the results? Were there any potential conflicts?      d. How have you managed them?      e. In the revised manuscript, in the Financial Disclosure section, discuss this and how you managed it. | - Thank you,  a. the funder has interest in publishing this paper. That why the institution support us during; study design, data collection and analysis.  b. It motivated the team.  c. the technical issues were handled by the authors, also, there is no protentional conflict of interest.  d. there was no protentional conflict of interest.  e. thank you, we have included this information under the disclosure section. |  |

**Reviewer 1**

|  | **Comments** | **Authors’ response** | **Page No.** |
| --- | --- | --- | --- |
| 1. | More or less comments were addressed. Please, your topic needs modification to make SMART. | - Thank you for your constructive feedback, we have shortened the topic from 28 words to 23 words, as per your suggestion. | #1 |
| 2 | Also, rewrite and cite your operational definitions. | - Thank you for your constructive comment, as per your suggestion we have made modification. | #9 |

**Reviewer 2**

|  | **Comments** | **Authors’ response** | **Page No.** |
| --- | --- | --- | --- |
| 1. | **Introduction**  The authors insist on using outdated statistics for the introduction. This is not an acceptable practice of international standards in the research community. It is important for the authors to note that they are writing the manuscript to inform current and future policy, but not past policy. It is therefore most appropriate to use current statistics. They should kindly address my earlier two comments on the introduction. | Thank you very much for your comment,  Initially we had organized the information concerning COVID-19 death report just before study period which was updated at a time (October 18, 2021), just following this period we are conducted our study from “October 10 to November 10, 2021”. | #3 |
| `2. | **Methods** Authors should kindly cite the scientific evidence used for the mean cut off point in the appropriate section of the manuscript. It is not enough to only write that on the response page. | - Thank you for your constructive comment, as per your suggestion we have cited the reference for cut off point on the operational definition parts. | #9 |
| 3. | **Results** Table 4 still contain odds ratios with different decimal points. Please make sure all decimal points are uniform (i.e. one decimal point or two decimal points for all). | - Thank you for your constructive comments, we have made changes as per your suggestion. | # table 4 |
| 4. | **Discussion** The authors claim that their findings are novel hence they cannot cite them. I do not agree that these novel findings. But even if these are novel findings, the reasons behind those findings MUST be accompanied by reasonable scientific evidence. So I urge the authors to kindly do the needful and cite appropriately. | - Thank you for your comment.  - In fact, we didn’t cite as you expected, the reason was, there were no single previous study reported on the variables “family size and occupation of pregnant women” which was significant in our study.  - As you know researcher or author can discuss (cite) only when he has got similar reports on the other studies. That is why we report our novel findings as follow:   - This study revealed a novel finding, that is, family size and occupation of pregnant women were found to have a significant association with WHO-recommended Covid 19 Prevention practice. Women with ≥10 family members were 96.2% less likely to have good Covid 19 preventive practice than those who had < 5 family members. This might be due to the fact that women with more family members are subjected to bear a lot of burdens to care for their families. Because of this, they may not give due attention to practice the recommended Covid 19 prevention practice. | #16 |
