# Supplementary material for: Determinants of WHO recommended COVID-19 prevention measures among pregnant women attending antenatal care during the third wave of COVID-19 in eastern Ethiopia, 2021
Source: PLoS One. 2023 May 25;18(5):e0284643. doi: 10.1371/journal.pone.0284643 (PMC10212107; doi:10.1371/journal.pone.0284643)
Supplement: Attachment — Submitted filename: Response to Reviewers.docx [file pone.0284643.s007.docx]

**Point-By-Point Feedback Based on Comments and Suggestions**

**Editor**

|  | **Comments** | **Authors’ response** | **Page No.** |
| --- | --- | --- | --- |
| 1. | Your responses to the Financial Disclosure query was inadequate. Based on the statement you provided in the financial disclosure, it needs to be clarified whether the authors' views, the institutional views, or both were reflected in the manuscript. Suppose the institution provided funds for the study and did not influence methods and participant selection, analysis, interpretation, and conclusion; it can be assumed that the views are of the authors and that there was no conflict of interest in the study. However, you stated that the funder, the institution in your particular case, played in all critical phases of the study – which shows it had a substantial influence in the study, leading to the assumption that there was a conflict of interest. If the statement was inserted by mistake, please revise it or state the institution's positions (including the formal statements, citations, or policy documents) about the study subject and how it impacted the findings, interpretations, and conclusion. | Thank you very much for pointing out this, we apologize for the confusion and we appreciate your view and we have rewrite it as per your suggestion. “This study was funded by Harar Health Science College. Funders play a role in funding the study and follow-up during the study process. The technical issues were handled by the authors, also, there was no protentional conflict of interest.”  However, Rose Ann Joyce Sagun Puetes said that “*Please note that funding information should not appear in the Acknowledgments section or other areas of your manuscript. We will only publish funding information present in the Funding Statement section of the online submission form. Please remove any funding-related text from the manuscript*.” We removed funding information from the manuscript | #18 |
| 2 | You added a new author to the manuscript. Please provide justifications and how the new author meets the authorship criteria. | - Thank you for your constructive comment, we have made changes on the author contribution section “AA, AD, and MY contributed to data analysis, drafting or revising the article. YA, LO and NA contributed drafting or revising the article and all authors have agreed on the journal to which the article will be submitted, gave final approval of the version to be published, and agree to be accountable for all aspects of the work.” | #18 |
| 3. | Page # I agree with Reviewer 2 that you should discuss the findings/ relationships between family size and occupation of pregnant women by leaving out the novelty concern, which is debatable. Your study had only 6 people in the “good” category, which is insufficient to make such a bold statement. Since you had a small sample size in that category, it could have been merged with the other category (5-9 people). Besides, Page# 13, lines 230-232, is unclear – the language needs improvement. Please rephrase it to convey the message. | - Thank you very much for your valuable comments, we have made changes as per your suggestion. | # 16 |
| 4 | In the abstract, methods section, discuss/ describe how you measured the outcome variable | - Thank you for your comment, the reason for not putting the measurement of outcome variable in the abstract due to word limitation, rather we have described in the body of manuscript under the method section “data collection tool and procedure” |  |
| 5. | Your manuscript needs proofreading and grammar check. | - Thank you for your constructive comment, as you have suggested, we have used Grammarly software, Ginger software and professional English language editors to recheck and correct the grammar and language flow. |  |
